# Author Correction: A tool for functional brain imaging with lifespan compliance

**DOI:** 10.1038/s41467-019-13752-8

**Published:** 2019-12-04

**Authors:** Ryan M. Hill, Elena Boto, Niall Holmes, Caroline Hartley, Zelekha A. Seedat, James Leggett, Gillian Roberts, Vishal Shah, Tim M. Tierney, Mark W. Woolrich, Charlotte J. Stagg, Gareth R. Barnes, Richard Bowtell, Rebeccah Slater, Matthew J. Brookes

**Affiliations:** 1 Sir Peter Mansfield Imaging Centre, School of Physics and Astronomy, University of Nottingham, University Park, Nottingham, NG7 2RD United Kingdom; 2 Department of Paediatrics, University of Oxford, John Radcliffe Hospital, Oxford, OX3 9DU United Kingdom; 3 QuSpin Inc., 2011 Cherry Street, Unit 112, Louisville, CO 80027 USA; 4 Wellcome Centre for Human Neuroimaging, UCL Institute of Neurology, University College London, 12 Queen Square, London, WC1N 3AR United Kingdom; 5 Oxford Centre for Human Brain Activity (OHBA), Wellcome Centre for Integrative Neuro-Imaging, Department of psychiatry, University of Oxford, Warneford Hospital, Oxford, OX3 7JX United Kingdom

**Keywords:** Magnetoencephalography, Biophysics, Development of the nervous system

Correction to: *Nature Communications* 10.1038/s41467-019-12486-x, published online 05 November 2019.

The original version of this Article contained an error in the spelling of the author Richard Bowtell, which was incorrectly given as Richard R. Bowtell. This has now been corrected in both the PDF and HTML versions of the Article.

