# Supplementary material for: A tool for functional brain imaging with lifespan compliance
Source: Nat Commun. 2019 Nov 5;10:4785. doi: 10.1038/s41467-019-12486-x (PMC6831615; doi:10.1038/s41467-019-12486-x)
Supplement: Supplementary file 1 — Supplementary Information [file 41467_2019_12486_MOESM1_ESM.pdf]

A tool for functional brain imaging with lifespan compliance: Supplementary Information

Ryan M. Hill, Elena Boto, Niall Holmes, Caroline Hartley, Zelekha A. Seedat, James Leggett, Gillian Roberts, Vishal Shah, Tim M. Tierney, Mark W. Woolrich, Charlotte J. Stagg, Gareth R. Barnes, Richard R. Bowtell, Rebeccah Slater, and Matthew J. Brookes

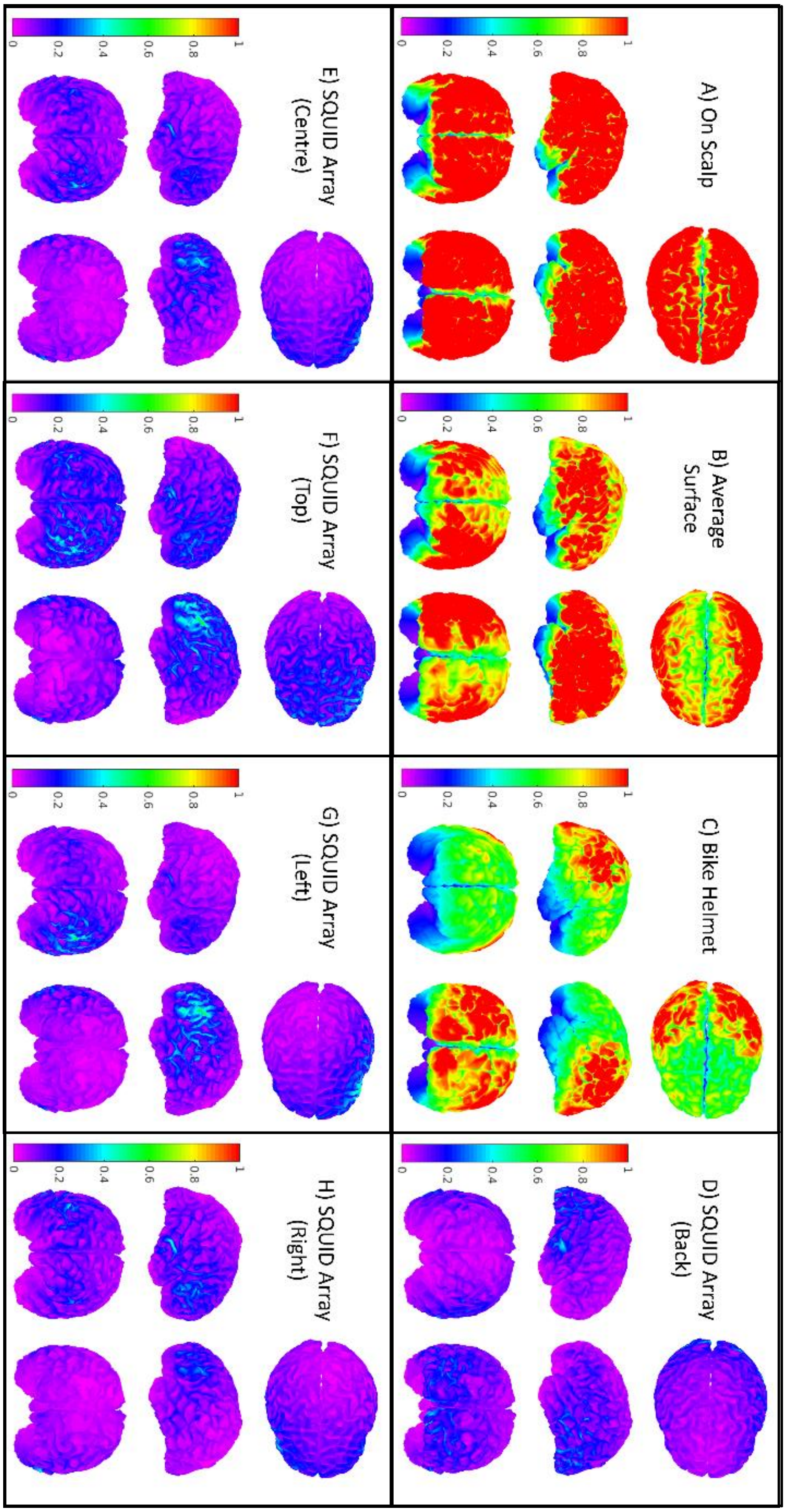

Supplementary Figure 1: Simulation results. *The same results as presented in figure 3 but from all sides of the brain for the three helmet designs (A-C), and for the SQUID array with the head in the centre of the array*, *as well as resting against each of the sides and the top of the CTF helmet* (D-H).
