# Supplementary material for: A tool for functional brain imaging with lifespan compliance
Source: Nat Commun. 2019 Nov 5;10:4785. doi: 10.1038/s41467-019-12486-x (PMC6831615; doi:10.1038/s41467-019-12486-x)
Supplement: Supplementary file 3 — Reporting Summary [file 41467_2019_12486_MOESM3_ESM.pdf]

nature research

Corresponding author(s): Matthew Brookes

Last updated by author(s): 29/08/2019

Reporting Summary

Nature Research wishes to improve the reproducibility of the work that we publish. This form provides structure for consistency and transparency in reporting. For further information on Nature Research policies, see Authors & Referees and the Editorial Policy Checklist.

Statistics

For all statistical analyses, confirm that the following items are present in the figure legend, table legend, main text, or Methods section.

n/a	Confirmed	
☐	☒	The exact sample size (*n*) for each experimental group/condition, given as a discrete number and unit of measurement
☐	☒	A statement on whether measurements were taken from distinct samples or whether the same sample was measured repeatedly
☒	☐	The statistical test(s) used AND whether they are one- or two-sided<br>*Only common tests should be described solely by name; describe more complex techniques in the Methods section.*
☒	☐	A description of all covariates tested
☒	☐	A description of any assumptions or corrections, such as tests of normality and adjustment for multiple comparisons
☒	☐	A full description of the statistical parameters including central tendency (e.g. means) or other basic estimates (e.g. regression coefficient) AND variation (e.g. standard deviation) or associated estimates of uncertainty (e.g. confidence intervals)
☒	☐	For null hypothesis testing, the test statistic (e.g. *F*, *t*, *r*) with confidence intervals, effect sizes, degrees of freedom and *P* value noted<br>*Give P values as exact values whenever suitable.*
☒	☐	For Bayesian analysis, information on the choice of priors and Markov chain Monte Carlo settings
☒	☐	For hierarchical and complex designs, identification of the appropriate level for tests and full reporting of outcomes
☒	☐	Estimates of effect sizes (e.g. Cohen's *d*, Pearson's *r*), indicating how they were calculated

Our web collection on statistics for biologists contains articles on many of the points above.

Software and code

Policy information about availability of computer code

Data collection	OPM data and channel triggers were collected using custom written software in LabView.<br>Motion tracking data were collected using Motive software by OptiTrack.
Data analysis	All data analysed using custom written software in MATLAB

For manuscripts utilizing custom algorithms or software that are central to the research but not yet described in published literature, software must be made available to editors/reviewers. We strongly encourage code deposition in a community repository (e.g. GitHub). See the Nature Research guidelines for submitting code & software for further information.

Data

Policy information about availability of data

All manuscripts must include a data availability statement. This statement should provide the following information, where applicable:

- Accession codes, unique identifiers, or web links for publicly available datasets
- A list of figures that have associated raw data
- A description of any restrictions on data availability

The datasets generated during and/or analysed during the current study are available from the corresponding author on request.

Field-specific reporting

Please select the one below that is the best fit for your research. If you are not sure, read the appropriate sections before making your selection.

☒ Life sciences ☐ Behavioural & social sciences ☐ Ecological, evolutionary & environmental sciences

For a reference copy of the document with all sections, see nature.com/documents/nr-reporting-summary-flat.pdf

Life sciences study design

All studies must disclose on these points even when the disclosure is negative.

Sample size

For the maternal touch, Robin Hood and Ukulele paradigms, they are proof of principle studies to demonstrate the ability to scan both paediatric and adult populations with a new type of brain scanner. In order to do this, 5 participants of different ages were chosen (ages 2-,5-,14- and two 24-years-old) to demonstrate lifespan compliance.
The finger abduction paradigm is used to demonstrate the ability to co-register MEG data to an anatomical MRI using a generic helmet to house the sensors. For this, 3 adult participants were scanned using the same rigid helmet to show the validity of our co-registration technique.

Data exclusions

Data from two sensors were removed from the maternal touch paradigm for the 2 year old participant. This is due to infrared light from the motion tracking camera interfering with only these two sensors. No other data were excluded.

Replication

Each paradigm was performed once for each subject per paradigm. In all cases, the expected changes in neural oscillations (i.e. beta desynchronisation during active period) were observed, and activity was localised to the expected regions of the brain (i.e. left sensorimotor cortex).

Randomization

We have no groups so this is not applicable.

Blinding

We have no groups so this is not applicable.

Reporting for specific materials, systems and methods

We require information from authors about some types of materials, experimental systems and methods used in many studies. Here, indicate whether each material, system or method listed is relevant to your study. If you are not sure if a list item applies to your research, read the appropriate section before selecting a response.

Materials & experimental systems

n/a	Involved in the study	
☒	☐	Antibodies
☒	☐	Eukaryotic cell lines
☒	☐	Palaeontology
☒	☐	Animals and other organisms
☐	☒	Human research participants
☒	☐	Clinical data

Methods

n/a	Involved in the study	
☒	☐	ChIP-seq
☒	☐	Flow cytometry
☒	☐	MRI-based neuroimaging

Human research participants

Policy information about studies involving human research participants

Population characteristics

For the maternal touch paradigm, three participants (all male) aged 2-, 5-, and 24-years-old took part.
For the Robin Hood paradigm, one female participant aged 14-years-old took part.
For the Ukulele paradigm, one female participant aged 24-years-old took part.
For the finger abduction task, two male participants aged 24 and 28 years and one female participant aged 27 years (all right handed).

Recruitment

Volunteers were recruited via posters placed around the university. The adult participants provided written informed consent (both to participation in the experiments, and to release of the photograph). Consent was given on behalf of the children by their parents (again to participation in the experiments and release of photographs).

Ethics oversight

University of Nottingham Medical School Ethics Committee

Note that full information on the approval of the study protocol must also be provided in the manuscript.
